# Cereal-legume intercropping: a smart review using topic modelling

**DOI:** 10.3389/fpls.2023.1228850

**Published:** 2024-01-08

**Authors:** Sofie Landschoot, Riccardo Zustovi, Kevin Dewitte, Nicola P. Randall, Steven Maenhout, Geert Haesaert

**Affiliations:** ^1^ Department of Plants and Crops, Faculty of BioScience Engineering, Ghent University, Ghent, Belgium; ^2^ Centre for Evidence-Based Agriculture, Harper Adams University, Newport, United Kingdom

**Keywords:** cereal, intercrop, legume, text mining, topic model

## Abstract

**Introduction:**

Over the last decade, there has been a growing interest in cereal-legume intercropping for sustainable agriculture. As a result numerous papers, including reviews, focus on this topic. Screening this large amount of papers, to identify knowledge gaps and future research opportunities, manually, would be a complex and time consuming task.

**Materials and methods:**

Bibliometric analysis combined with text mining and topic modelling, to automatically find topics and to derive a representation of intercropping papers as a potential solution to reduce the workload was tested. Both common (e.g. wheat and soybean) as well as underutilized crops (e.g. buckwheat, lupin, triticale) were the focus of this study. The corpus used for the analysis was retrieved from Web of Science and Scopus on 5*^th^* September 2022 and consisted of 4,732 papers.

**Results:**

The number of papers on cereal-legume intercropping increased in recent years, with most studies being located in China. Literature mainly dealt with the cereals maize and wheat and the legume soybean whereas buckwheat and lupin received little attention from academic researchers. These underutilized crops are certainly interesting to be used as intercropping partners, however, additional research on optimization of management and cultivar’s choice is important. Yield and nitrogen fixation are the most commonly studied traits in cereal-legume intercropping. Last decade, there is an increasing interest in climate resilience, sustainability and biodiversity. Also the term “ecosystem services” came into play, but still with a low frequency. The regulating services and provisioning services seem to be the most studied, in contrast terms related to potential cultural services were not encountered.

**Discussion:**

In conclusion, based on this review several research opportunities were identified. Minor crops like lupin and buckwheat need to be evaluated for their role as intercropping partners. The interaction between species based on e.g. root exudates needs to be further unraveled. Also diseases, pests and weeds in relation to intercropping deserve more attention and finally more in-depth research on the additional benefits/ecosystem services associated with intercropping systems is necessary.

## 1 Introduction

Intensive monoculture has, over the years, resulted in a higher productivity at the expense of decreasing levels of biodiversity. Moreover, monocultures of identical plants cause damage to the ecosystem, especially when combined with blanket application of fertilizers and pesticides (Bourke et al., 2021). Currently, there is a growing interest in moving away from monocultures to more diverse cropping systems. Intercropping, the simultaneous cultivation of two or more crops on a given piece of land, is one method of crop diversification. Combining a cereal and legume is by far the most common type of intercropping and an important route towards sustainable intensification (Gaba et al., 2015; Verret et al., 2020). Legumes establish symbiosis with certain soil bacteria, collectively known as rhizobia, which are capable of fixing atmospheric nitrogen and can thus be grown with minimal fertilizer inputs (Quinones et al., 2022). Well-managed legume-based intercopping systems are uniquely positioned to curtail the existential challenge posed by climate change through the significant contribution that legumes can make towards limiting green house gas emissions, reducing pesticide use, minimizing fertilizer losses, reversing biodiversity declines, and delivering secure and resilient food systems (Iannetta et al., 2021).

Increased sustainable crop productivity is among the most important and most frequently cited benefit of intercropping (Bybee-Finley and Ryan, 2018). Next to the crop production/food provisioning services, intercropping is also associated with the alleviation of other ecosystem services, including improving soil and water quality, controlling pests, and mitigating climate change (Brooker et al., 2015; Gaba et al., 2015; Daryanto et al., 2020). Despite these well-known benefits, intercropping remains uncommon in most modern farming systems (Iannetta et al., 2021; Meunier et al., 2022). In order to be able to compete with large scale monocultures, resource use efficiency and crop yield in intercropping systems must be optimized (Li et al., 2013; Li et al., 2014). To optimally exploit the possible benefits associated with intercropping systems, a deeper insight into several aspects of intercropping is necessary. Firstly, a greater understanding of crop production and cultivation methods is required including basic agronomy e.g. tillage methods, optimal seed rates, crop and varieties combinations, plant nutrition, harvest methods, processing facilities and market demand for intercrops harvested simultaneously (Kumar et al., 2012; Holt et al., 2021; Meunier et al., 2022). Secondly, a more in-depth research on the mechanisms of interactions between crop genotypes and species, for example, enhanced resource availability through niche complementarity, is necessary. Demie et al. (2022) reviewed the effect of genotype in cereal-legume intercropping. It was concluded that in most cases a significant genotype × cropping system interaction was present, revealing the importance of genotype choice. To further unravel the interactions in intercropping systems (genotype × genotype × environment × management), a comprehensive study on a wide range of phenological and morphological traits including plant height, maturity, root growth, soil nutrient and water acquisition, and diseases, among others, which is lacking hitherto, is necessary. In addition, intercropping systems are important for resilience and long-term stability of agricultural systems. Ecological advances include better understanding of the context-dependency of interactions, the mechanisms behind disease and pest avoidance, the links between above- and below-ground systems, and the role of microtopographic variation in coexistence (Brooker et al., 2015).

Although a lot of research on intercropping has already been published and subsequently used as basis for various reviews (Brooker et al., 2015; Ananthi et al., 2017; Daryanto et al., 2020; Yin et al., 2020; Demie et al., 2022), meta-analyses (Seran and Brintha, 2010; Himmelstein et al., 2017; Raseduzzaman and Steen Jensen, 2017; Reiss and Drinkwater, 2018; Rodriguez et al., 2020; Tang et al., 2021) and bibliometric studies (Lv et al., 2021; Feng et al., 2022), additional effort is necessary to gain deeper insight into the numerous publications and the current research trends in intercropping. Topic modelling has proven itself as a useful tool for exploratory analysis of a large number of papers (Evans, 2014). However, it has rarely been applied in the context of an exploratory literature review in the domain of agriculture. Topic modelling allows emerging patterns in data to be discovered, thereby also allowing the identification of novel knowledge. In the present study, to the best of our knowledge, this is the first time topic modelling in combination with various text mining techniques has been applied to screen literature related to legumecereal intercropping.

This study is part of the H2020 CROPDIVA project(Grant agreement ID: 101000847), where one aspect focusses on diverse cropping systems and cultivation techniques for triticale, oat, hulless-barley, buckwheat, lupin and faba bean. The CROPDIVA project also aims to go beyond the current state-of-the art and thus to close knowledge gaps related to intercropping systems. To identify directions for future research, this study applied bibliometrics, text mining and topic modelling. The main themes studied in research on legume-cereal intercropping were mapped and knowledge gaps were identified. More specifically the following research questions were addressed:

- Which are the main crops studied in legume-cereal intercropping systems?
- Which, potentially interesting, species combinations are understudied?
- Which ecosystem services are mainly addressed?

## 2 Materials and methods

**Figure 1:**
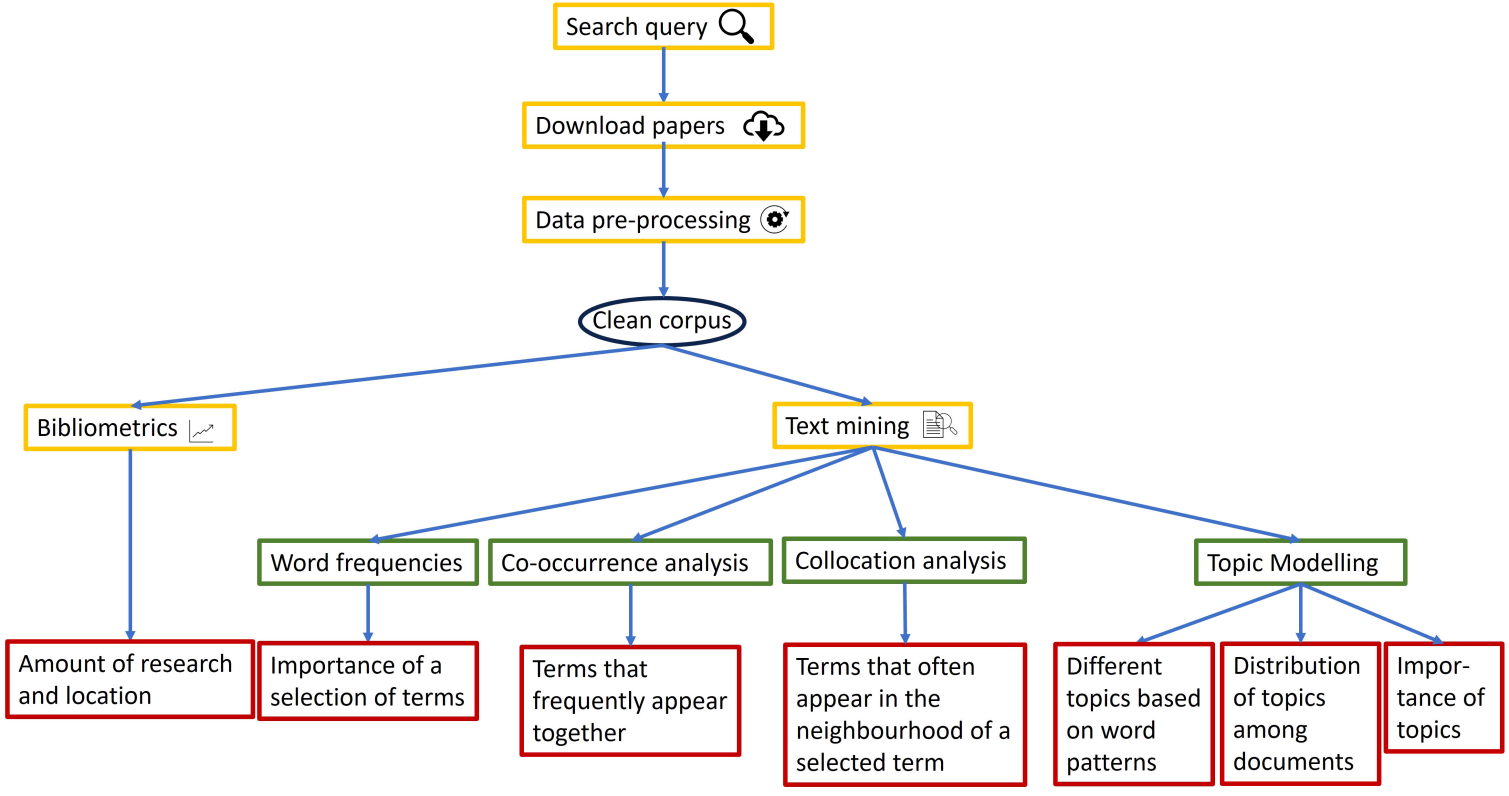
Research roadmap with the different steps and methods used, together with the insights offered by the various approaches (red boxes).

The various steps and methods to analyze the literature are described below and are illustrated in **Figure 1**.

### 2.1 Literature screening and bibliometric analysis

A systematic search for peer-reviewed publications on legume-cereal intercropping was performed by querying the databases of Web of Science and Scopus on 5*^th^* September 2022. Our search query contained the six above-mentioned crops together with the main cereal crops wheat, maize, the main grain legumes soybean and pea, and synonyms for intercropping. In addition, papers in the field of (agro)forestry were excluded as this is beyond the scope of the project. The following search string was applied on the title of the paper, the key words and the abstract:

(“mixed crop*” OR intercrop* OR “mixed intercrop*” OR (“mixed cult” AND crop) OR “mixed stand*”) AND (lupin* OR “faba bean*” OR oat* OR wheat* OR barley* OR buckwheat* OR triticale* OR soybean* OR pea* OR maize) NOT (forest* OR agroforest*).

The full records (title, keywords, abstract, author information, journal information, publisher) of the retrieved references were saved as.txt files and duplicates were removed. This collection of texts is called a “corpus”, which is large and unstructured set of texts.

### 2.2 Data pre-processing

Before the retrieved corpus could be analyzed, data pre-processing was necessary as each text contains a certain fraction of irrelevant information that blurs the informative content to some extent. Firstly, the texts were converted to lower-case and punctuation, stop words (and, or, the etc.) and numbers were removed. The latter were removed since for this analysis the quantified results (e.g. yields) are not studied. Also bigrams and trigrams, two or three consecutive words that are more likely to co-occur rather than appear separately, were identified and concatenated into one string to optimize interpretability of the topics. Some examples of bigrams and trigrams are e.g. faba bean, cover crop, Land Equivalent Ratio. In addition, stemming, using Porter’s algorithm (Porter, 1980) was performed, i.e. each word was replaced by its base word (stem). Finally spare terms, which do not help to differentiate between topics, were removed.

### 2.3 Bibliometric analysis

Bibliometric analysis is a scientific computer-assisted method of bibliographic counting that can uncover trends in evolutionary nuances of a specific field, while shedding light on the emerging areas in that field. More specifically, it can be used to assess journal performance (e.g. evolution of impact factor), collaboration patterns, and research constituents, and to explore the intellectual structure of a specific domain in the extant literature (Donthu et al., 2021). Here, a bibliometric analysis was used to study the annual trends and the geographical trends in the selected titles and abstracts of publications relating to intercropping.

### 2.4 Text mining

#### 2.4.1 Concept

Text mining is the application of techniques from machine learning and computational statistics to find useful patterns in text data. There are many different approaches to analyze text data (Holzinger et al., 2014). The two most popular techniques are counting frequencies and topic modelling. These techniques consider each text as a simple bag of words and pay little attention to its structure. In contrast, the study of co-occurrences focuses on collocations of words inside of a text (Bourgeois et al., 2015).

#### 2.4.2 Word frequencies

Calculation of word frequencies is the most used and the most popular text mining method and consists of the computation of the frequencies (or number of occurrences) for a selection of words. In this study the dynamics for a selection of words over time was analyzed. To better visualize the dynamics, these data were subjected to loess regression, a non-parametric approach that fits multiple regressions in a local neighborhood (Cleveland et al., 1992).

#### 2.4.3 Co-occurrence analysis

Since this study deals with intercropping, an agronomic practice where two or more crops are grown on the same field, it was of particular interest to gain insight in which crops occur frequently together. Thus for each given word *p*, and for each other word *w*, it was counted how many times *w* is present in a text which contains *p*. The results of this analysis are represented as heatmaps.

#### 2.4.4 Collocation analysis

To gain insight into which ecosystem services are studied, a collocation analysis was done. Collocation is a term used to describe words that co-occur (significantly) more often together than would be expected by chance. Unlike bigrams and trigrams, words that collocate do not have to be immediately adjacent but can also encompass several slots. The collocation strength was calculated according to Wiedemann and Niekler (2017).

#### 2.4.5 Topic modelling

**Figure 2:**
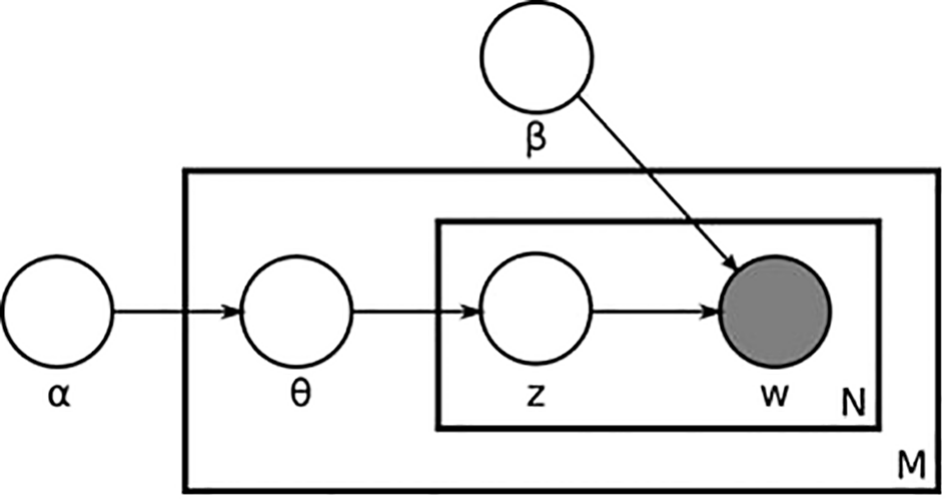
Plate notation representing the Latent Dirichlet Allocation (LDA) algorithm. The outer plate represents documents, while the inner plate represents the repeated choice of topics and words within a document. Area in *M* denotes the number of documents. *N* is the number of words in a given document. *α* is the parameter of the Dirichlet prior on the per-document topic distributions. *β* is the parameter of the Dirichlet prior to the per-topic word distribution, *θ*m is the topic distribution for document *m*, *z_mn_* is the topic for the *n*-th word in document *m*, *w_mn_* is the specific word (Blei et al., 2003).

Topic modelling is an unsupervised machine learning technique, that seeks to find hidden semantic structure in text documents and is an efficient method to analyze large volumes of articles. Topics can be conceived of as networks of collocation terms that, because of the co-occurrence across documents, can be assumed to refer to the same semantic domain (or topic). This assumes that, if a document is about a certain topic, one would expect words, that are related to that topic, to appear in the document more often than in documents dealing with other topics (Chaney and Blei, 2021). Latent Dirichlet Allocation (LDA) is one of the most popular algorithms in topic modelling. It was initially proposed by Pritchard et al. (2000) and then improved by Blei et al. (2003). For LDA, it is considered that data instances are being generated from a latent process, which is dependent on hidden variable. In **Figure 2** the dependencies among the various LDA parameters are given. High *α* indicates that each document is likely to contain a mixture of most of the topics (not just one or two), whereas low *α* indicates each document will likely contain just a few of topics. High *β* indicates that each topic will contain a mixture of most of the words, whereas low *β* indicates the topic has a low mixture of words.

LDA algorithm assumes that new documents are created in the following way:

1. Determine the number of words in document.
2. Choose a topic mixture for the document over a fixed set of topics (example: 20% topic A, 50% topic B, 30% topic C).
3. Generate the words in the document by:
 • pick a topic based on the document’s multinomial distribution.
 [*z_m,n_* ∼ Multinomial(*θ_m_*)]
 • pick a word based on topic’s multinomial distribution.
 [*w_m,n_* ∼ Multinomial(*ϕ_zmn_*)] (where *ϕ_zmn_* is the word distribution for topic *z*)
4. Repeat the process for *n* number of iteration until the distribution of the words in the topics meet the criteria (step 2).

To run this model, the number of topics *K* needs to be defined. To find a “good” number of topics, four different metrics were calculated: Arun2010 (Arun et al., 2010) and CaoJuan2009 (Cao et al., 2009) which need to be minimized, and Griffiths2004 (Griffiths and Steyvers, 2004) and Deveaud2014 (Deveaud et al., 2014) which need to be maximized. These measures select the best number of topics using a symmetric Kullback-Leibler divergence of salient distributions which are derived from the factorization of the document-term matrix (Vargas et al., 2021).

**Figure 3:**
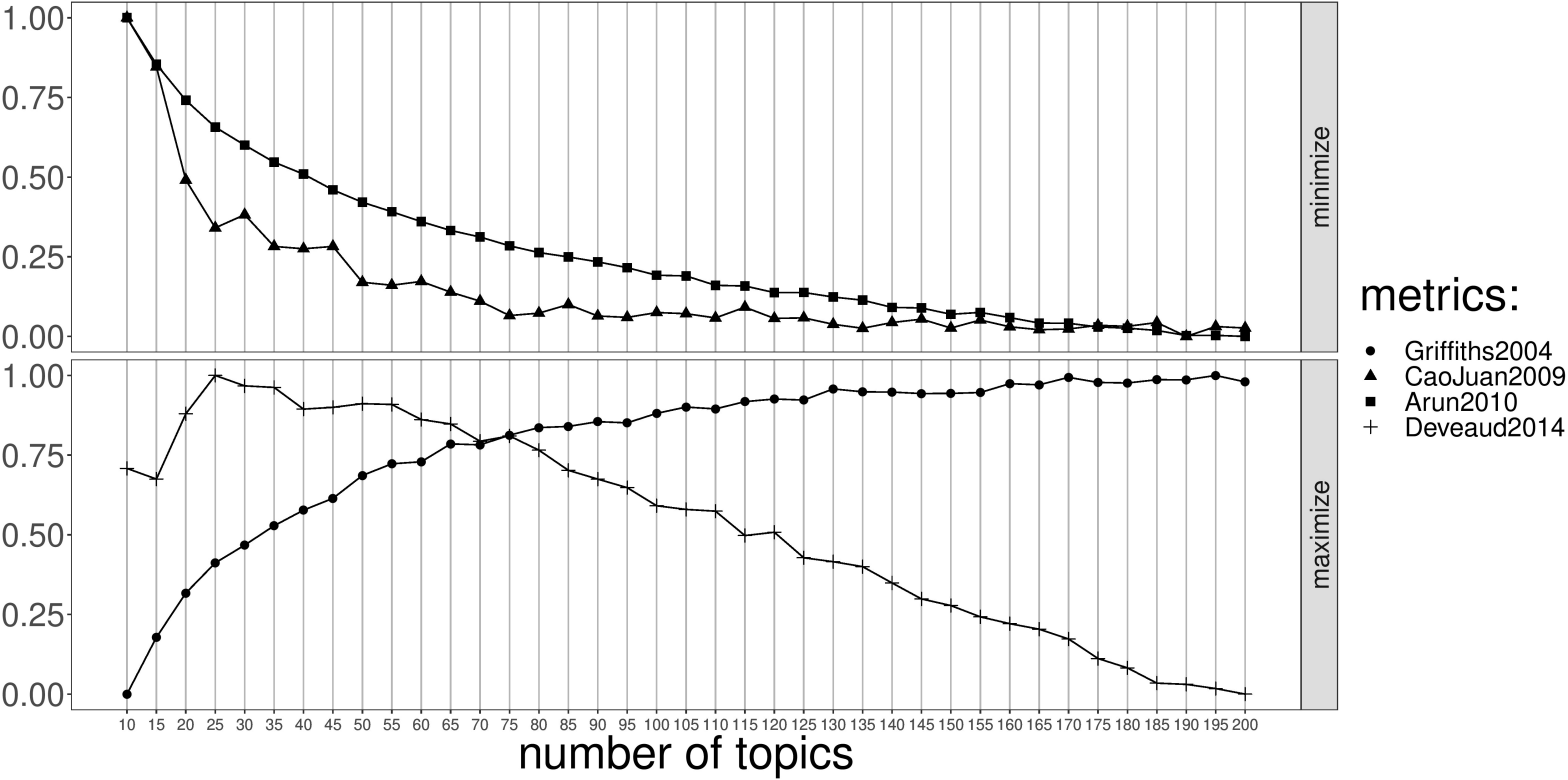
Value of the performance measures (Griffiths2004, CaoJuan2009, Arun2010 and Deveaud2014) (y-axis) in function of the number of topics (x-axis).

**Figure 3** shows the calculated metrics, to goal is to find the point at which the various metrics are either minimized (in the case of the top two metrics) or maximized (in the case of the bottom two). For this particular corpus Deveaud2014 suggests 25 topics, while the metrics Arun20210, CaoJuan2009 and Griffits2004 continue to decrease/increase as the number of topics increases. Therefore, the optimal number of topics was set at 150, the point at which there is no clear gain by increasing the number of topics.

### 2.5 Hierarchical clustering

The latent topics from LDA are assumed to be independent of each other, which may not be accurate in case of co-occurring topics. For instance, the terms “maize” and “soybean” were part of several topics which are very likely related to each other. So, it is possible that there are a couple of overlapping topics. To see which topics should be grouped, a hierarchical clustering model with wardD clustering (Murtagh and Legendre, 2014) was performed using the Hellinger distance (distance between two probability vectors).

### 2.6 Software

All analyses were performed with R (R Core Team, 2022). The bibliometric analysis was done with the R-package “Bibliometrix” (Aria and Cuccurullo, 2017). The packages “tm” (Feinerer et al., 2008) and “textclean” (Rinker, 2018) were used for data pre-processing. The packages “tidytext” (Silge and Robinson, 2016), “ldatuning” (Murzintcev, 2016) and “topicmodels” (Grün and Hornik, 2011) were used for topic modelling. Clustering was done with the “hclust” function from the stats packages and “ggplot2” (Wickham, 2009) was loaded for result visualization.

## 3 Results

### 3.1 Bibliometric analysis

**Figure 4:**
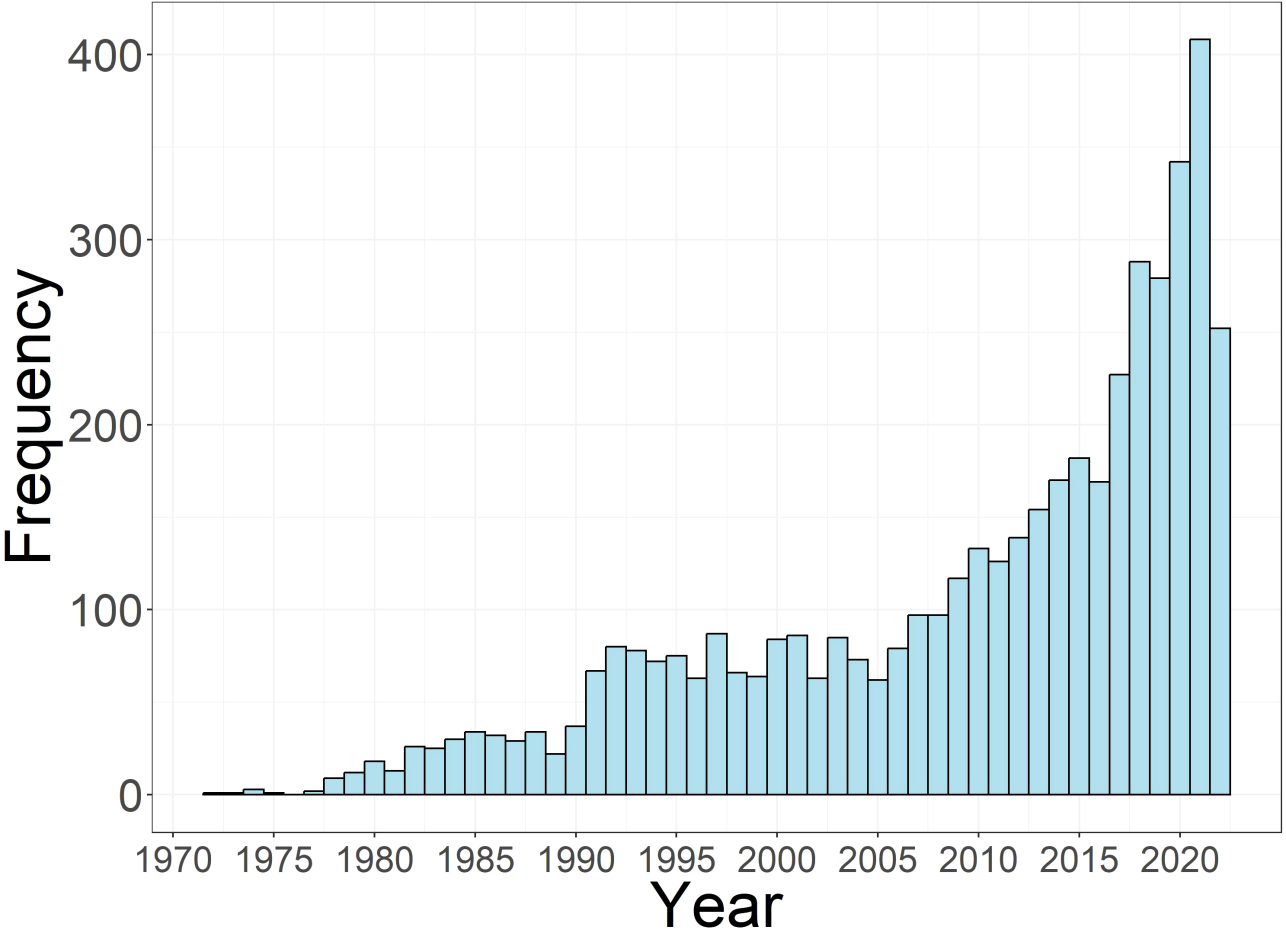
Temporal distribution of the number of published papers retrieved from a Web of Science/Scopus search for intercropping and the ten named crops (lupin, faba bean, oat, wheat, barley, buckwheat, triticale, soybean, pea, maize) on 5*^th^* September 2022.

In total, the searches from the two databases (Web of Science and Scopus) resulted in a total of 4,732 papers, after duplicate removal. An increasing trend in the number of papers published was registered, with a steep increasing starting from 2017 (**Figure 4**).

**Figure 5:**
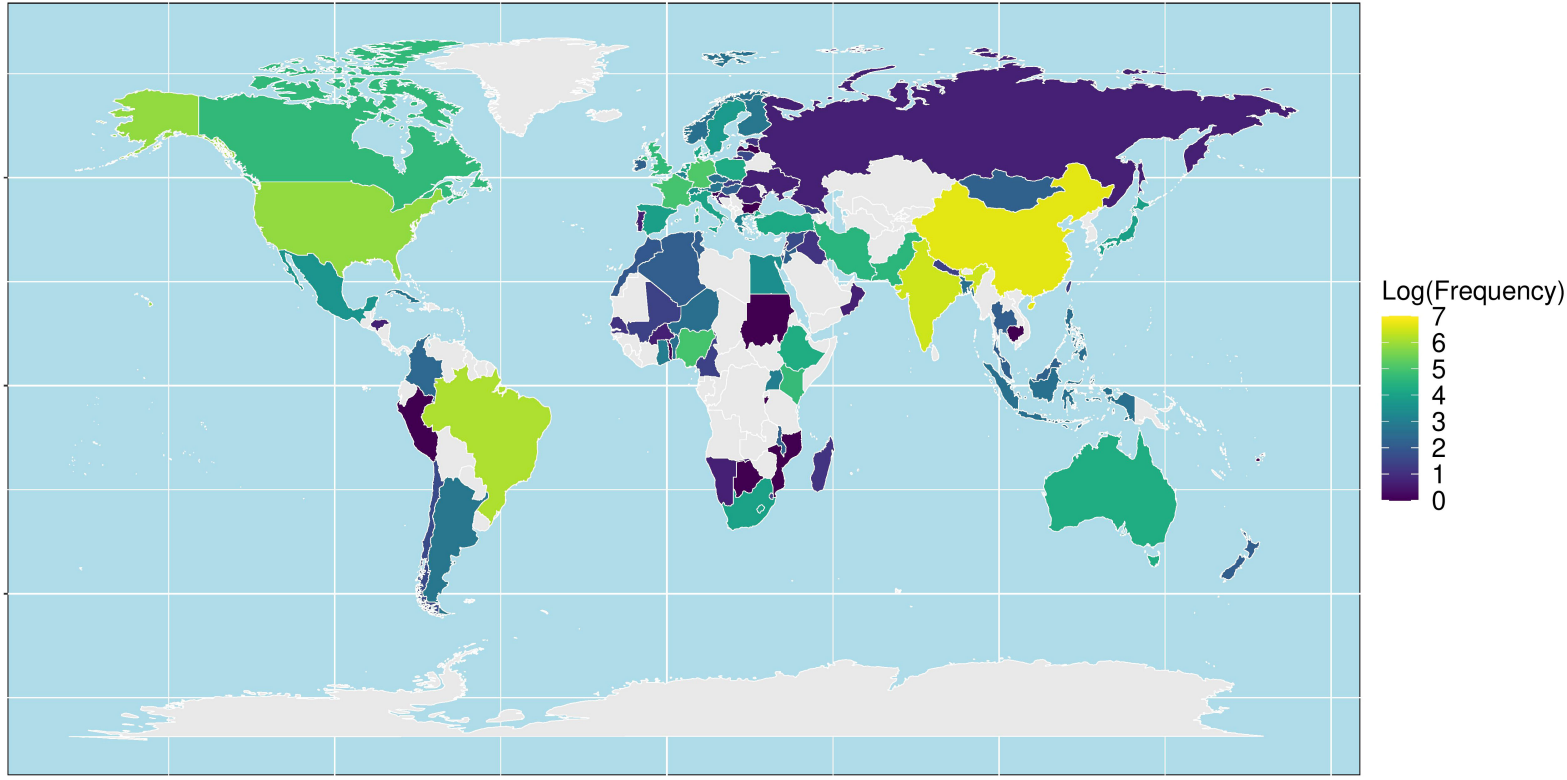
World distribution of the number of published papers, based on the corresponding author country, retrieved from a Web of Science/Scopus search for intercropping and the ten named crops (lupin, faba bean, oat, wheat, barley, buckwheat, triticale, soybean, pea, maize) on 5*^th^* September 2022.

**Figure 5** illustrates the worldwide distribution of the included studies. The most studies containing the intercropping terms were conducted in Asia, mainly in China (785) and in India (626). The number of included European studies was comparable to the number of studies originating from the Americas with 943 and 1003 publications, respectively. In the Americas, Brazil (456) and the USA (345) were the most publishing countries. In Europe, most studies are from Germany and France, 163 and 141 studies, respectively.

Although all 4,732 papers were included in the topic modelling, it is likely that some of the included papers were not relevant to the question. We tried to find the best balance between sensitivity (retrieve all relevant documents by using a broad search) and specificity (retrieve only relevant documents in a small precise search) in our search query, however, it has been shown that this is very challenging (Bramer et al., 2018).

### 3.2 Text mining

**Figure 6:**
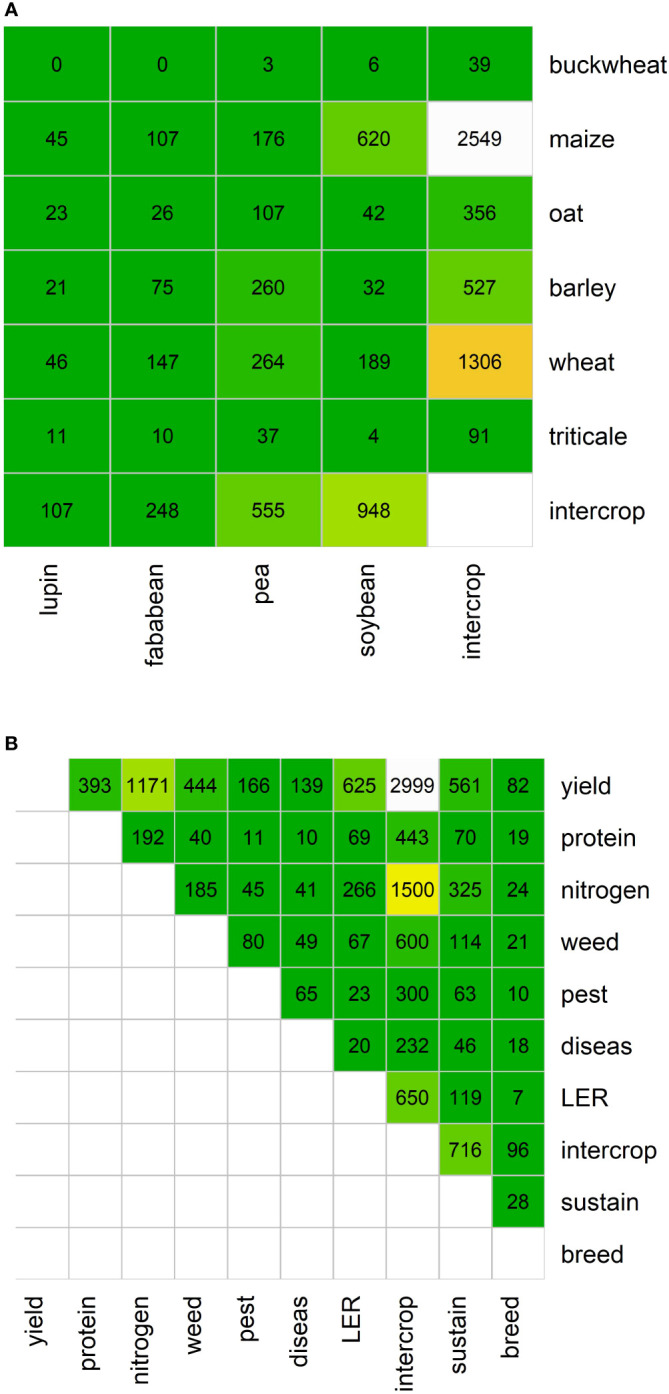
**(A)** Heatmap showing the co-occurrence of named cereals and legumes in papers collated from searches of intercropping related terms. **(B)** Heatmap showing the co-occurrence of various traits related to intercropping. Colors correspond with cell values, from white (high) to orange (moderate) to green (low).

To gain a first insight into the selected papers, word co-occurrences were calculated by computing how two words occur together in the corpus. Focusing on the co-occurrences of the selected cereals and legumes in the intercropping papers (**Figure 6A**), it can be seen that maize is the main crop reported alongside intercropping, closely followed by wheat. Buckwheat is the least frequently reported crop with respect to the co-occurrence of a (pseudo)cereal and a legume, maize and soybean seem to be the most popular combination. Wheat and pea are also frequently reported together. However, it should be noted that if two crops frequently appear together in papers, this does not automatically mean that they are intercropping partners. Furthermore, there are no studies mentioning both buckwheat and lupin. Focusing on the traits that were studied (**Figure 6B**), yield and nitrogen appear to receive considerable attention. Also sustainability seems to be important, whereas e.g. diseases and pests were less mentioned in literature. The Land Equivalent Ratio (LER) was identified in 650 papers (14%), indicating that besides this measure to evaluate the performance of intercropping systems also other metrics are used (Zustovi et al., 2023).

**Figure 7:**
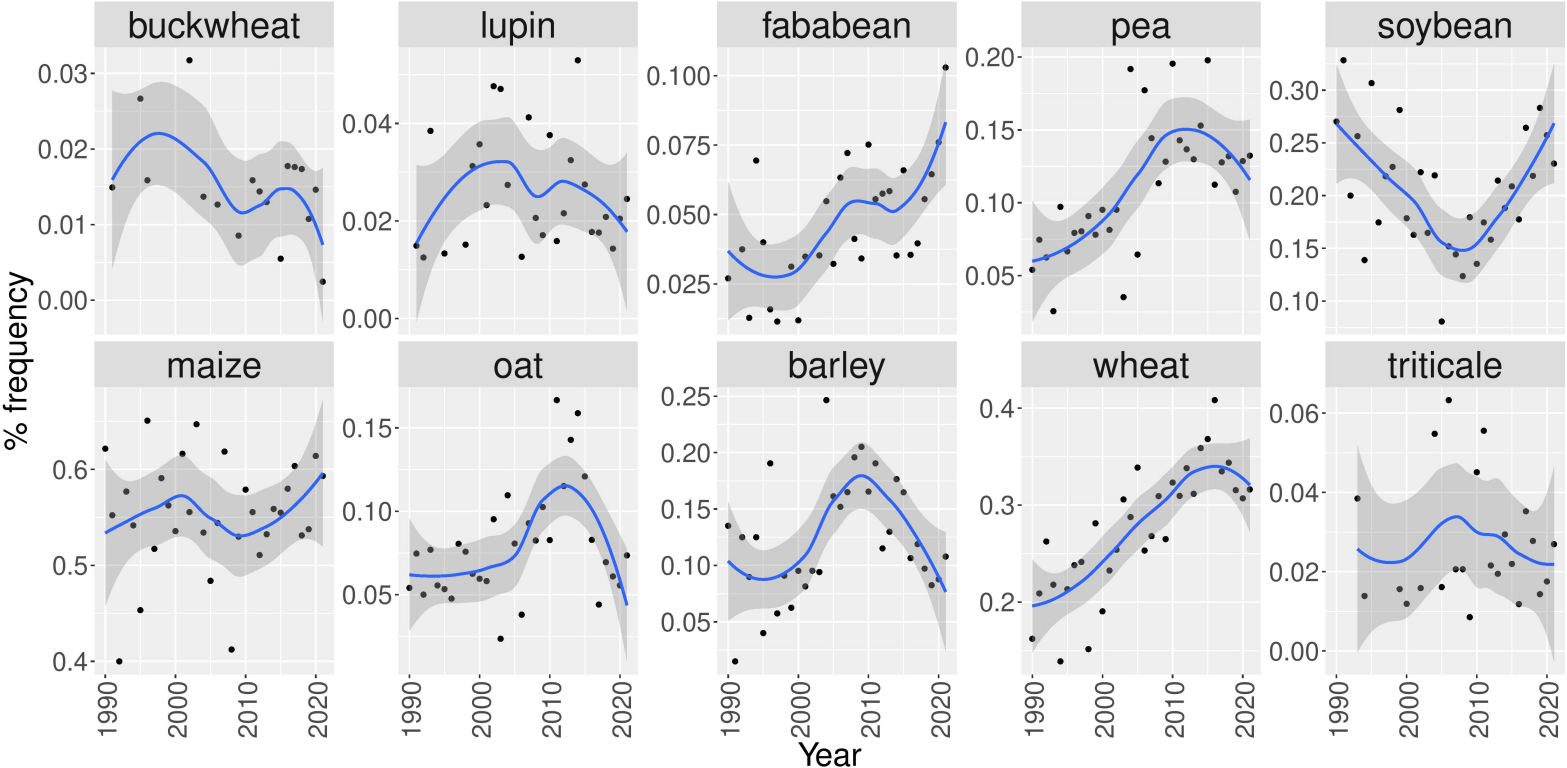
Relative frequency of retrieved papers containing the terms “buckwheat”, ”lupin”, ”faba bean”, ”pea”, ”soybean”, ”maize”, ”oat”, ”barley”, ”wheat” or “triticale” from 1990 until 2021. The blue line represents the loess regression and the grey area is the 95% confidence interval.

The presence of the crops mentioned in the papers changes over time (**Figure 7**). Based on the absolute counts for all crops, with the notable exception of buckwheat, the frequency of occurrence of all preselected crops demonstrate an increasing trend over the last 30 years (**Supplementary Figure S1**). This can be explained by the fact that the number of publications in the corpus clearly increased over time (**Figure 4**), but the relative frequencies show a more crop-specific picture. For the main cereal crops, i.e. maize and wheat, there is an increasing trend over the entire study period, with approximately 50% of the most recent papers referring to one of these crops. For the other crops publication rates fluctuates over time. More recently, there seems to be a renewed interest in the protein crops faba bean and soybean, whereas the share of pea and lupin in relevant searches decreases. After an increasing trend until 2010 the frequency of the cereals barley and oat in intercropping research has decreased over the last few years. However, it should be noted that in absolute frequency, these crops remain popular subjects of intercropping research papers.

**Figure 8:**
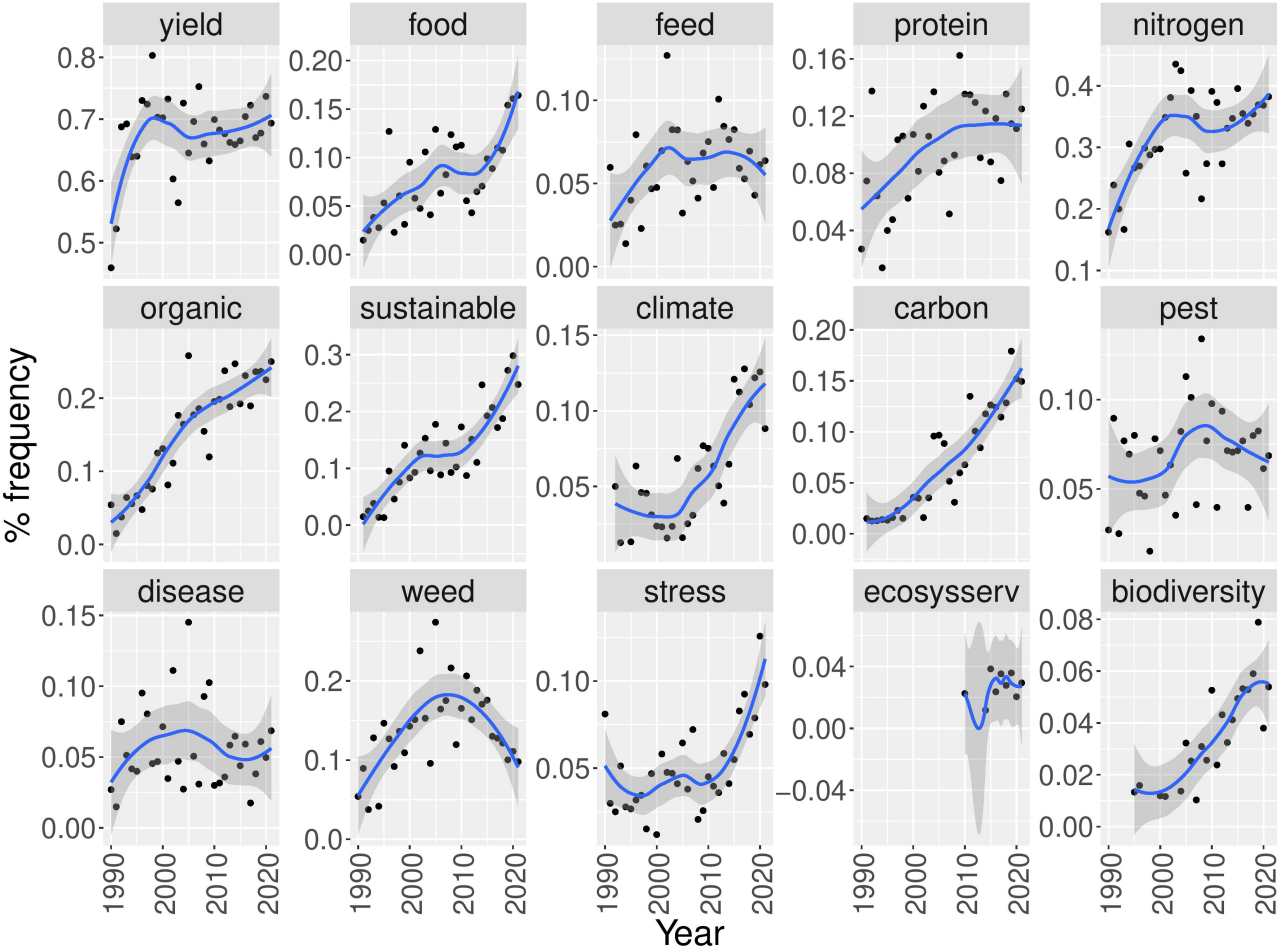
Relative frequency of documents containing various research topics related to intercropping from 1990 until 2021. The blue line represents the loess regression and the grey area is the 95% confidence interval.

Similar to the absolute counts of crop occurrences, the trend of all examined trait occurrences demonstrates a steep increase over the last years as a result of the increasing number of publications devoted to intercropping (**Supplementary Figure S2**). Based on the relative frequencies (**Figure 8**), it can be seen that the interest in food applications seems to have increased during the last few years while applications in feed are becoming less attractive as study topics. Also disease, pest and weed reduction appear to have never been extensively studied in relation to intercropping. In contrast, the relative frequency of terms related to climate, sustainability and biodiversity is on the rise. Finally, the term “ecosystem service” has been introduced in intercropping literature since 2010 (**Figure 8**).

### 3.3 Collocation analysis

**Figure 9:**
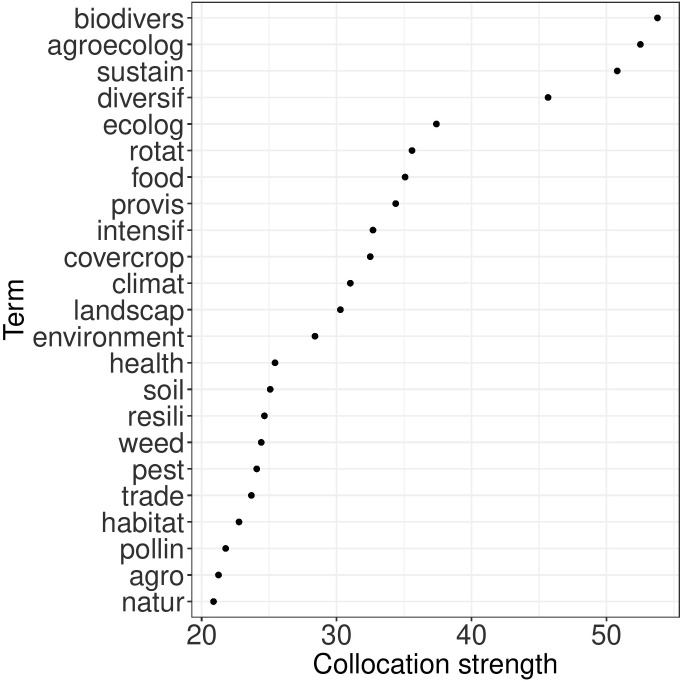
Collocation strengths (x-axis) of the terms (y-axis) most strongly associated with “ecosystem service”.

A collocation analysis was performed to gain insight into the various ecosystem services that are studied in relation to intercropping (**Figure 9**). It can be seen that terms biodiversity, agro-ecology, sustainability, diversification and ecology are most strongly associated with “ecosystem service”. Food and provisioning services come second, while other terms like health, pollinators, habitat which are related to the regulation services, are also frequently associated with “ecosystem service”. Terms related to cultural services such as recreation, aesthetic values, spiritual values, education, tourism, do not appear to be associated as intercropping services.

### 3.4 Topic modelling

**Figure 10:**
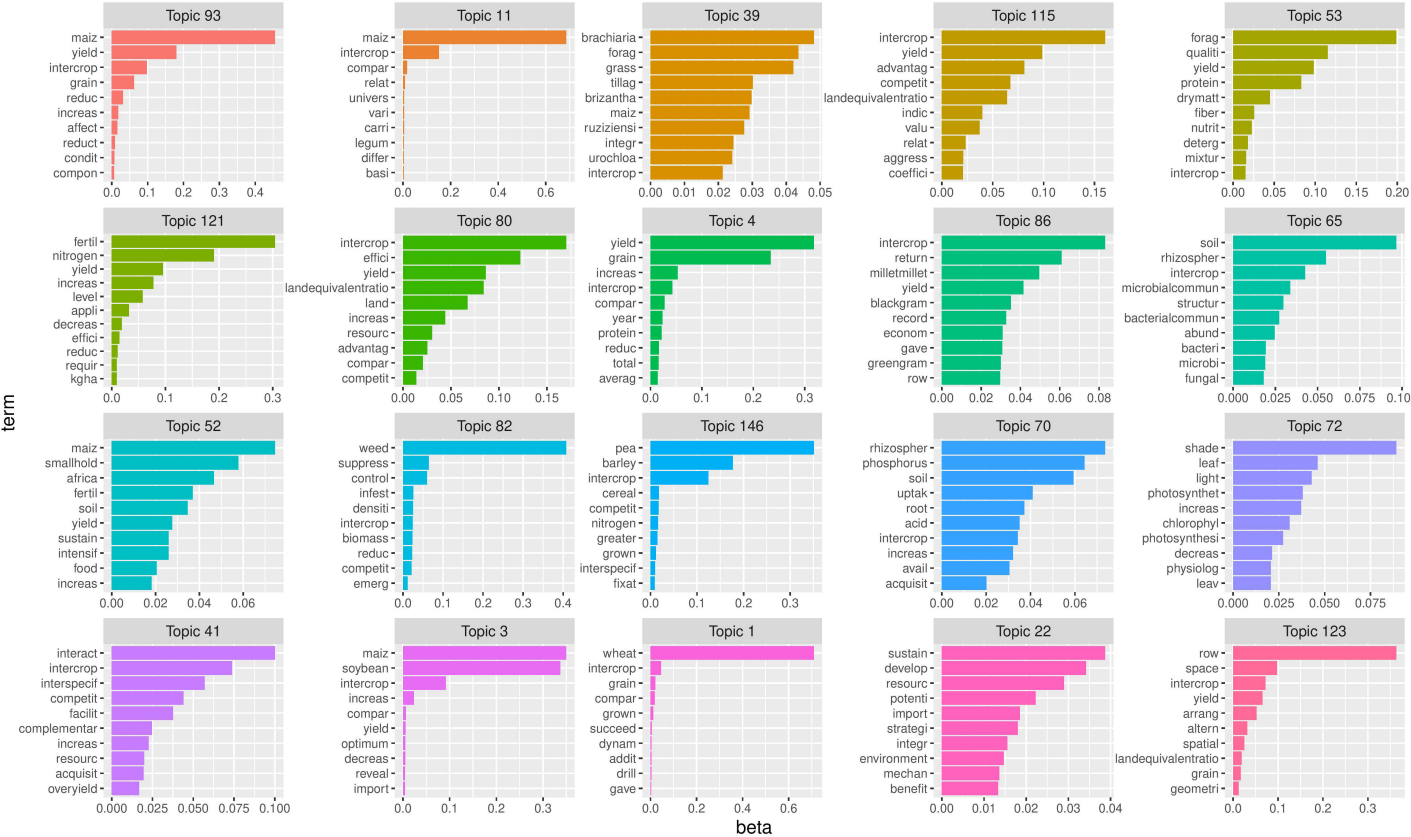
Twenty topics with the top ten terms ordered according to their importance. Remark that the topic numbers on top are the numbers generated by the algorithm and are not related to the importance of the topic. On the X-axis the posterior word probabilities of the top 10 terms(Y-axis). The longer the bar, the higher the probability that the corresponding term will appear in a document dealing with this particular topic. Remark that due to stemming the words on the Y-axis are reduced to their stem.

In **Figure 10** the top ten terms of top 20 topics are given, where the topics are sorted according to their probability within the entire collection. Remark that the numbers of the topics in **Figure 10** are not related to their importance, but generated randomly by the algorithm since LDA begins with random assignment of topics to each word and iteratively improves the assignment of topics to words through Gibbs sampling. It can be seen that most abstracts in this corpus contain the terms “maize”, “soybean”, “intercrop” and “yield”. This illustrates the importance of both crops in intercropping literature. “Pea” and “barley” are the main terms in topic 146 and “wheat” is the top term of topic 1. It can also be noted that underutilized crops e.g. lupin, buckwheat and even triticale never occur in the top 20 terms of intercropping literature.

In addition, it can be noted that some topics are perhaps not relevant to our work, but may have just been included as intercropping related terms may have been referred to at some point. e.g. topic 86 deals mainly with the crops millet, greengram and blackgram, which were not subject of this study, also the grasses and forages mentioned in topic 39 are not relevant for this study. As mentioned in Section 3.1, this is due to the sensitivity/specificity issue.

### 3.5 Hierarchical clustering of topics

**Figure 11:**
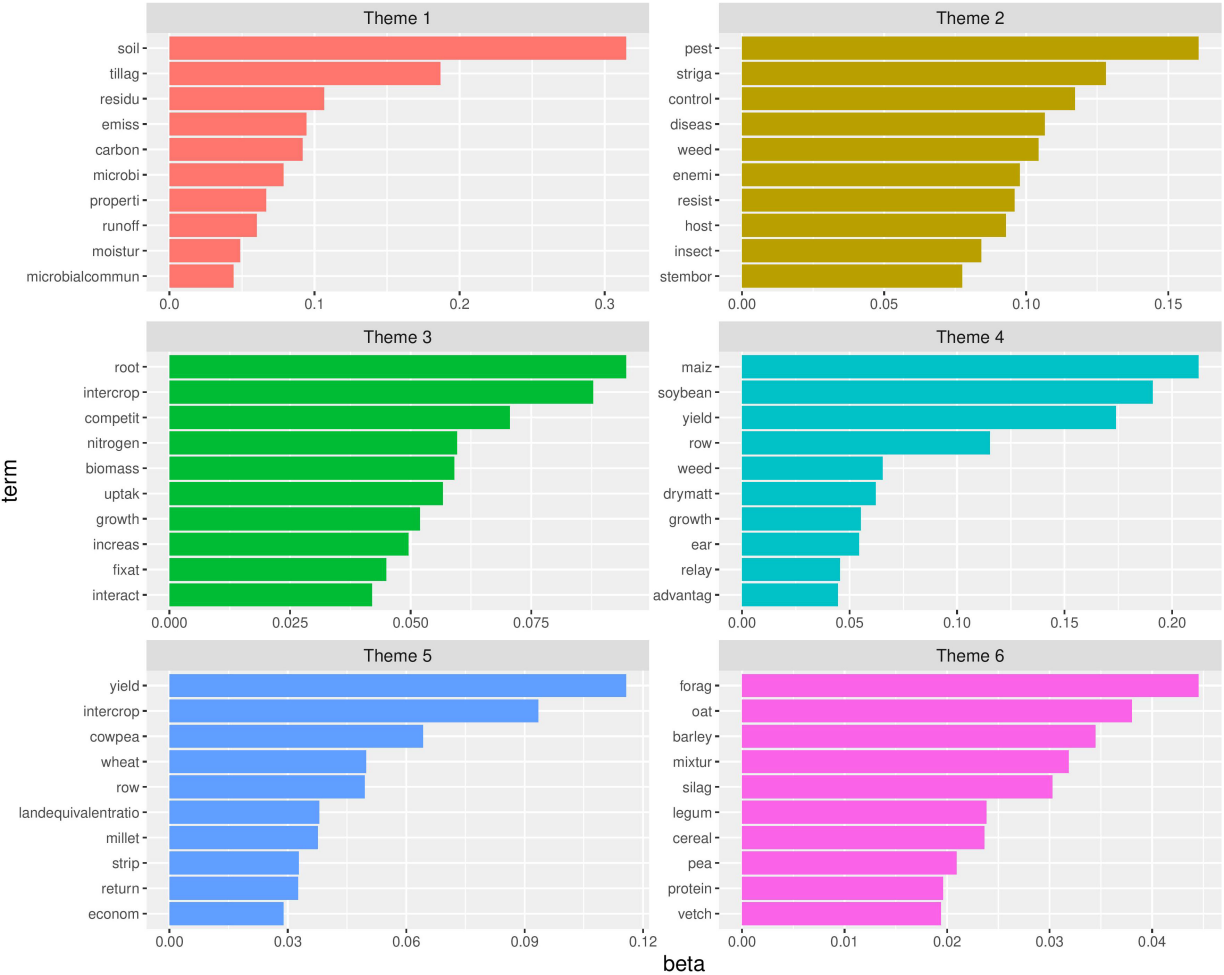
Six themes with the top ten terms ordered according to their frequency. On the X-axis the posterior word probabilities of the top 10 terms (Y-axis). The longer the bar, the more probability the corresponding term has to belong to that theme. Remark that due to stemming the words on the Y-axis are reduced to their stem.

It can be seen that there is a certain degree of overlap between the major keywords of various topics as, for example, topics 3, 11 and 93 all have maize as their most probable term (**Figure 10**). To gain further insight in their relatedness, the topics were clustered hierarchically using the Hellinger distance as a (dis)similarity measure. Based on the hierarchical clustering (**Supplementary Figure S4**), it was decided that the topics could be clustered into to six major themes, that could be characterized by the posterior probabilities the words they include (**Figure 11**).

## 4 Discussion

Manual exploratory literature reviews is a time-consuming process, with limited processing power, resulting in a low number of papers analyzed (Asmussen and Moller, 2019). Recently, bibliometric analyses have been applied in the field of intercropping (Lv et al., 2021; Feng et al., 2022). While bibliometric analyses allow to elucidate the major trends in any particular field of research, they generally only scratch the surface in terms of domain-specific knowledge gaps and future directions of research. Topic modelling surpasses bibliometric analyses and enables more detailed analyses to be made (Ozyurt and Ayaz, 2022). Until now, few applications of topic modelling have been used on research papers, especially in the domain of agriculture. In this study, topic modelling was used to sift through the ever expanding body of scientific literature on intercropping, in order to gain insight into the main topics that are being studied and to identify possible research gaps in this domain.

A dedicated search query was constructed to retrieve the titles and abstracts of 4,732 papers on cereal-legume intercropping systems that involve the six (oat, hulless barley, faba bean, buckwheat, lupin and triticale) CROPDIVA crops. Over the last decade, intercropping has been rediscovered by scientific research, as reflected by a steep increase in the number of published papers that include this topic. Intercropping is indeed receiving increasing global interest as an agricultural practice as farmers strive to be more sustainable and maintain soil health (Glaze-Corcoran et al., 2020). The analysis of the geographical distribution revealed that most papers came from China. This can be explained by the fact that intercropping has been practiced in China for thousands of years, but also by the renewed interest of Chinese researchers in this cropping system as a way to introduce a more ecological and sustainable form of agriculture (Knoerzer et al., 2009). While intercropping is also widespread throughout small holder farming systems in tropical African regions (Lithourgidis et al., 2011), the fraction of scientific papers originating from Africa was rather relatively small. This can be partially explained by the fact that our search query consisted of crops that are mainly grown in non-tropical regions.

The main species being used in cereal-legume intercropping are maize and wheat with soybean and pea as their most popular legume partners, respectively. Intercropping of these major crops offers many advantages compared to sole cropping (Blessing et al., 2022) and both crops are already widely grown. These combinations thus do not contribute to the demand for increasing crop diversification. Triticale, lupin and buckwheat are, on the other hand, hardly encountered in intercropping studies. Therefore, incorporating a greater diversity of crop types, including orphan crops, enables an increase in production efficiency through facilitation and complementary mechanisms to deliver multiple functional and resource-use benefits over more traditional cropping systems (Iannetta et al., 2021). In contrast to e.g. wheat, buckwheat and triticale do not require high agricultural inputs and can be grown on marginal lands. These crops can thus be considered as promising sustainable intercrop partners (Davis-Knight and Weightman, 2008; Chrungoo and Chettry, 2021). The European orphan crop lupin is a protein crop that is considered as an optimal alternative to soybean cultivation in temperate climates with acidic soils. Among grain legumes, lupin exhibits both the most variable yield and the least competitive ability against weeds, meaning that intercropping winter white lupin for grain may have a higher potential of development compared the growing lupin in a pure culture (Carton et al., 2020). According to Yan et al. (2020) buckwheat is a suitable intercrop for reducing pollen-mediated gene flow from genetically modified cotton, and may be useful for reducing pollen-mediated gene flow from other insect-pollinated genetically modified crops, while simultaneously contributing to control of specific insect pests due to attraction of their natural enemies.

The crop combination of buckwheat and lupin seems to be a promising addition to the intercropping landscape which justifies its detailed evaluation in the CROPDIVA project. However, before proposing and popularizing lupin and buckwheat in intercropping systems, additional research is necessary. The currently cultivated gene pools of orphan crops still contain variation in important interaction traits because this diversity has not been lost through monoculture breeding as is the case for the elite cultivars of major crops (Kamenya et al., 2021; Kumar et al., 2023). This implies that there are significant opportunities for designing effective intercrop systems involving these underutilized crops. This depends of course on suitable breeding methods being made available, a topic we return to below (Dawson et al., 2019).

Concerning the studied aspects in intercropping systems, it was observed that yield is by far the most important trait studied. Weed, disease and pest suppression are also important traits, but do not receive a lot attention in intercropping studies. The importance of climate resilience, sustainability and biodiversity aspects related to intercropping exponentially increases last years. In addition, intercropping traits involved in technical, quality and other downstream processes including ripening time and seed color require further optimization. Furthermore, also complementary aspects related to species synergy during the growth period, for example, “mixing ability” and “species compatibility” are equally important breeding goals that, to date, have received little attention in scientific literature (Kiaer et al., 2022).

Recently, the term “ecosystem services” has been introduced in scientific literature devoted to intercropping. From the collocation analysis it was clear that the benefits from intercropping are mostly discussed in the context of sustainability and biodiversity, which can be categorized under supporting services. Also terms related to the provisioning services (e.g. food, feed, fiber) and regulating services (e.g. climate regulation, pollination of crops, carbon storage) were encountered. In contrast, cultural services (intellectual inspiration and recreational environment) seem to be not, or rarely studied as a service resulting from the intercropping systems.

Land Equivalent Ratio (LER) was reported in 14% of the studies for assessing the relative response of intercropping compared with mono-cultures. The LER is an index that describes the relative land area required to grow the same quantity of both crop species in the mixture (species 1 and 2), if grown as monocultures rather than as mixtures. Furthermore, LER should be used in combination with other measures to adequately assess the relative advantages of intercropping in terms of productivity and value (Zustovi et al., 2023), e.g. if a monocropping system requires more external resources (e.g., nitrogen, weed suppression), LER smaller than unity might not be disadvantageous (Khanal et al., 2021).

Topic modelling has learned that intercropping literature can be divided into 150 different topics, which can be clustered into to six major themes. The terms in three of these themes were related to specific crops frequently co-occurring in intercropping literature, e.g. maize and soybean, wheat and pea, oat and barley together with the traits studied, yield, protein and forage. The other three themes consisted of terms related to the potential advantages of intercropping e.g. carbon storage, microbial communities, emissions and disease, weed, pest control and root interactions, nitrogen fixation. Topic modelling also allows to identify papers that are strongly associated with one or more research topics. Furthermore, analysis of the topics that occur at low frequencies allows to identify potential knowledge gaps with respect to the CROPDIVA project. Based on this analysis several knowledge gaps that need further exploration are encountered. Very few papers study lupin and buckwheat, so the potential of these crops as intercropping partners needs to be screened in various settings. In addition, more attention has to go to diseases, pests and weeds in relation to intercropping both possible avoidance strategies as well as control measures. Also root exudates in relation to nodulation should be further explored together with the potential of inoculation with various Rhizobium strains. The wider benefits of intercropping in terms of ecosystem services should also be addressed.

It has been demonstrated that bibliometrics combined with text mining and topic modelling provide an added value to manual screening of literature. However, there are also some limitations associated with this approach to screen literature. Automatic literature screening is often characterized by a high sensitivity and low specificity (Zhang et al., 2022). Although the search query was designed to find a good balance between sensitivity and specificity, also papers that are not relevant for our study were retrieved by this query and reversely, some papers have likely slipped through the cracks of the query. It has been shown that automatic literature screening systems can reach sensitivity as high as 95%, despite at the expense of specificity, since reviewers try to include every publication relevant the topic of review (Zhang et al., 2022). Interpreting the topics from the topic modelling can be difficult due to a lack of context, understanding of how the words are used in the context is necessary. Topic modelling can help to identify patterns in a corpus, but it is no substitute for human interpretation of a text.

## 5 Conclusions

This study illustrates that bibliometrics combined with text mining and topic modelling allows to efficiently screen a large number of research papers, yielding interesting insights and conclusions. Intercropping has been rediscovered by the scientific community and studies are conducted all over the world. Although there is a growing recognition of the role of orphan crops in maintaining biodiversity, they are still underused in intercropping systems, which currently mainly focus on maize, soybean and wheat. Studying how orphan crops can be improved and introduced in intercropping systems will contribute to a sustainable crop production and increased diversity. Yield, the provisioning service, remains the most important trait studied in intercropping. However, next to food, feed production also regulating, cultural and supporting services must be taken into account in evaluating intercropping systems.

## Data availability statement

The original contributions presented in the study are included in the article/**Supplementary Material**. Further inquiries can be directed to the corresponding author.

## Author contributions

SL and NR: conceptualization and methodology; SL and SM: data analysis; SL, RZ, and SM: writing and editing; SL, RZ, KD, NR, SM, and GH: reviewed and editing; SM and GH: supervision. All authors contributed to the article and approved the submitted version.
