# Supplementary material for: Cereal-legume intercropping: a smart review using topic modelling
Source: Front Plant Sci. 2024 Jan 8;14:1228850. doi: 10.3389/fpls.2023.1228850 (PMC10800527; doi:10.3389/fpls.2023.1228850)
Supplement: Supplementary file 1 [file DataSheet_1.pdf]

# Supplementary Material

## 1 SUPPLEMENTARY DATA

Supplementary Material should be uploaded separately on submission. Please include any supplementary data, figures and/or tables. All supplementary files are deposited to FigShare for permanent storage and receive a DOI.

Supplementary material is not typeset so please ensure that all information is clearly presented, the appropriate caption is included in the file and not in the manuscript, and that the style conforms to the rest of the article. To avoid discrepancies between the published article and the supplementary material, please do not add the title, author list, affiliations or correspondence in the supplementary files.

## 2 SUPPLEMENTARY TABLES AND FIGURES

For more information on Supplementary Material and for details on the different file types accepted, please see the Supplementary Material section of the Author Guidelines.

Figures, tables, and images will be published under a Creative Commons CC-BY licence and permission must be obtained for use of copyrighted material from other sources (including re-published/adapted/modified/partial figures and images from the internet). It is the responsibility of the authors to acquire the licenses, to follow any citation instructions requested by third-party rights holders, and cover any supplementary charges.

### 2.1 Figures

Figure S3 depicts the document to topics probabilities for a random selection of three documents, where the x–axes are the probabilities and the y-axes are the topic numbers. Some papers belong almost exclusively to one particular topic (e.g., document 2018 to topic 14) while other documents are admixtures of different topics. In contrast e.g., documents 1507 en 2994 belong partially to different identified topics. These document to topic probabilities allow to identify the topics that have low overall probability of occurrence in the corpus. The topics with the lowest average gamma and that are related to the CROPDIVA project are topic 85 with the main terms ”lupin”, ”white” and ”intercrop”, topic 101 with the terms ”sourc”, ”buckwheat” and ”wild”, topic 2 with the terms ”nodul”, ”nitrogen”, ”fixat”, ”exud” and topic 141 with the terms ”inocul”, ”growth”, ”bacteria” and ”rhizobia”.

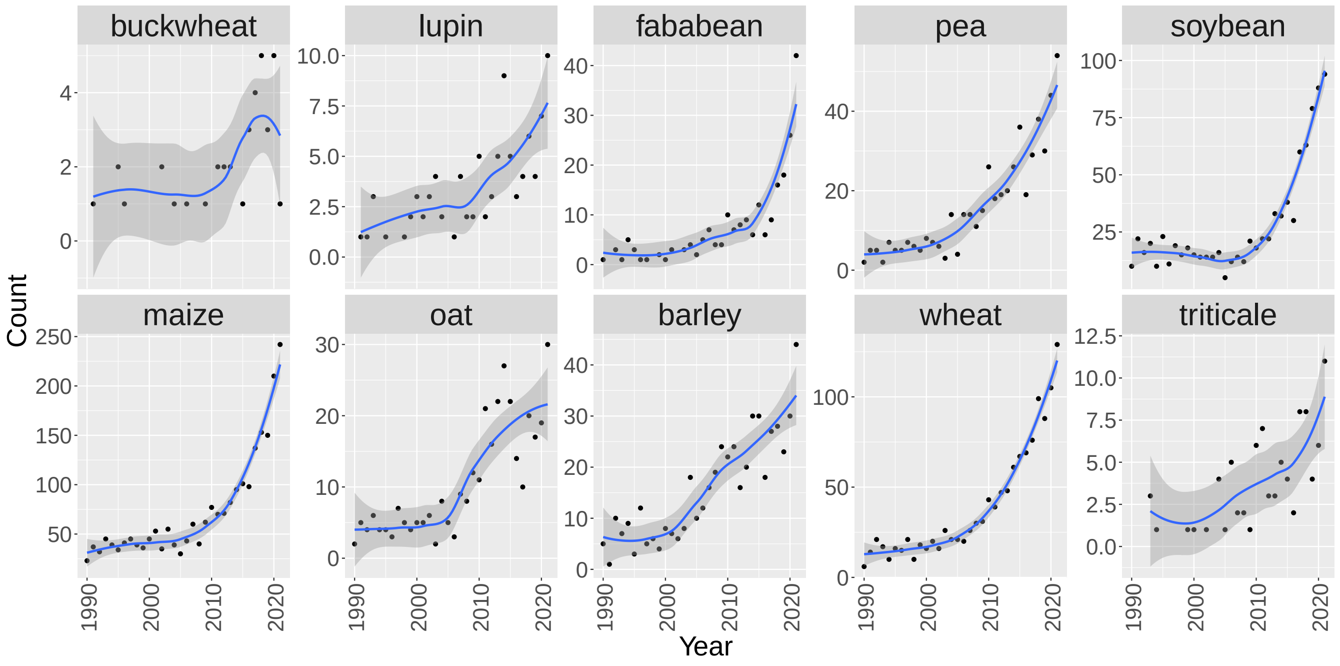


**Figure S1.** Absolute frequency of retrieved papers containing the terms ”buckwheat”,”lupin”,”faba bean”,”pea”,”soybean”,”’maize”’,”oat”,”barley”,”wheat” or ”triticale” from 1990 until 2021. The blue line represents the loess regression and the grey area is the 95,% confidence interval.

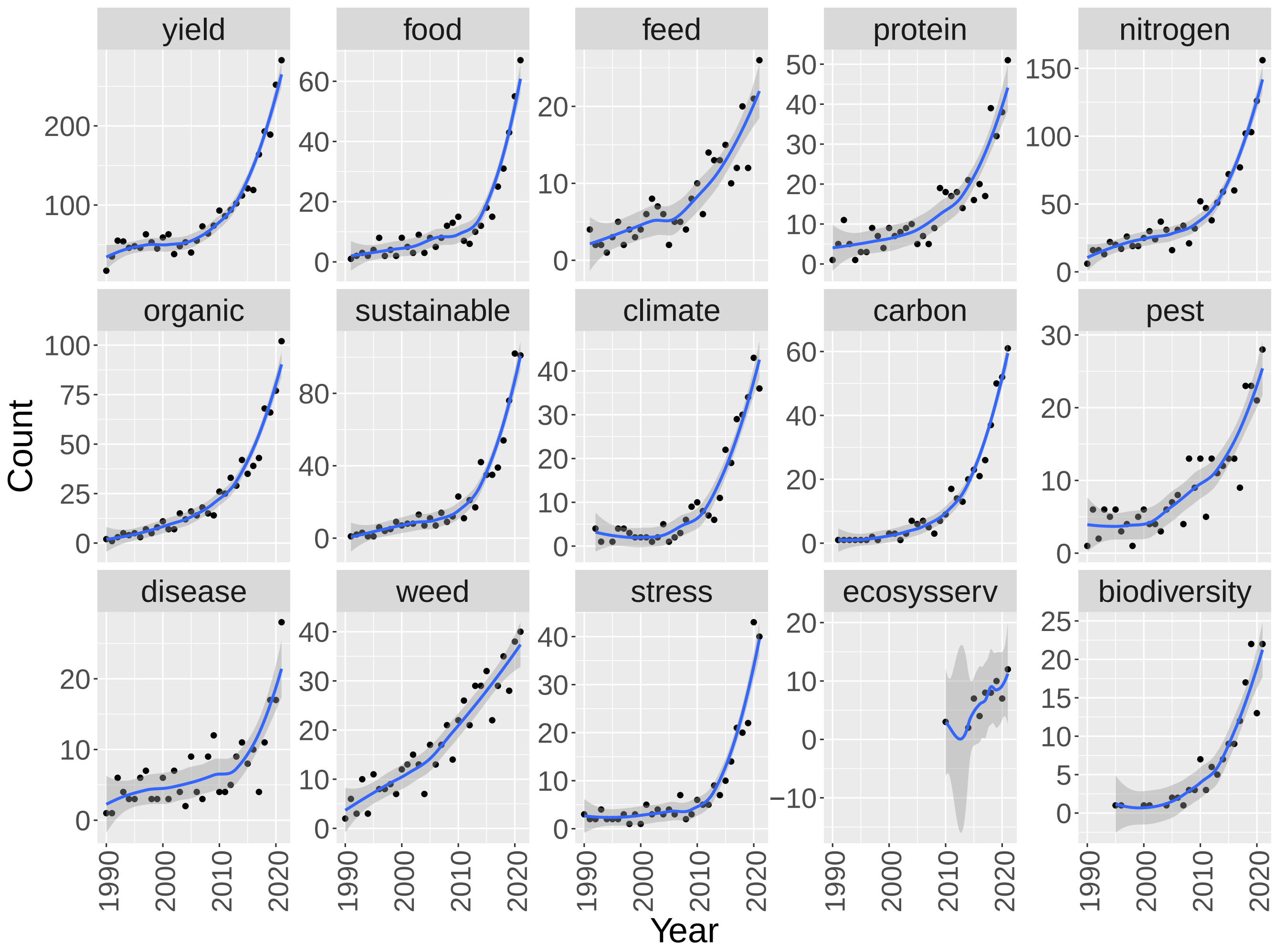


**Figure S2.** Relative frequency of documents containing various research topics related to intercropping from 1990 until 2021. The blue line represents the loess regression and the grey area is the confidence interval.

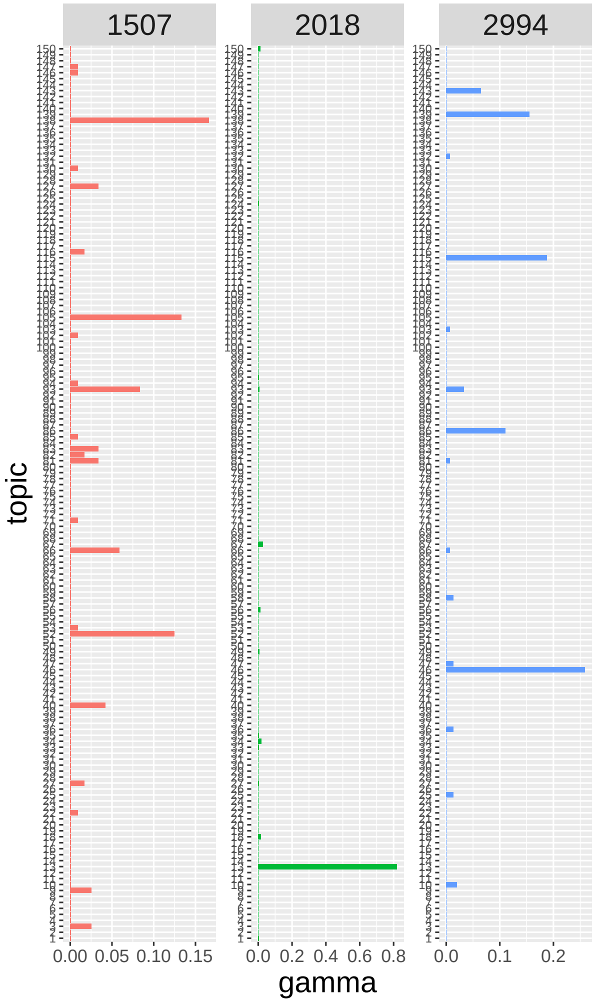


**Figure S3.** Document to topic probabilities.

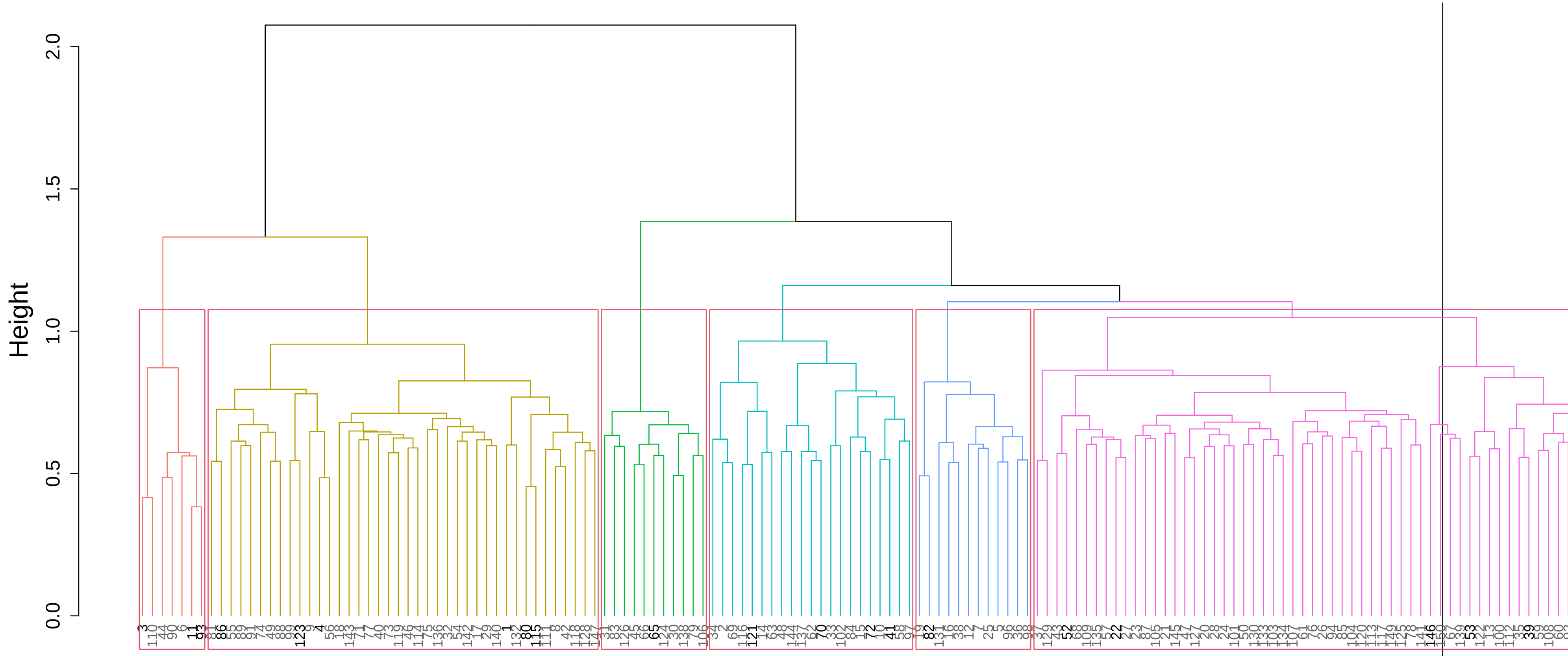


**Figure S4.** Hierarchical clustering dendrogram of the 150 LDA topics. The dendrogram was cut into six clusters. The node labels denote the LDA topic numbers. The Y-axis shows the distance between the clusters as computed with Hellinger distance. The main 20 topics shown in Figure **??** are coloured in black and the remaining topics are coloured in grey.
